# Unphysiological lung strain promotes ventilation-induced lung injury via activation of the PECAM-1/Src/STAT3 signaling pathway

**DOI:** 10.3389/fphar.2024.1469783

**Published:** 2025-01-08

**Authors:** Gang Liu, Bin-Bin Dong, Zi-Heng Ding, Chao Lan, Chang-Ju Zhu, Qi Liu

**Affiliations:** ^1^ Department of Emergency Intensive Care Unit, The First Affiliated Hospital of Zhengzhou University, Zhengzhou, Henan, China; ^2^ Translational Medicine Center, The First Affiliated Hospital of Zhengzhou University, Zhengzhou, Henan, China; ^3^ Department of Emergency, The First Affiliated Hospital of Zhengzhou University, Zhengzhou, Henan, China

**Keywords:** ventilation-induced lung injury, unphysiological lung strain, PECAM-1, Src/STAT3, pyroptosis

## Abstract

**Introduction:**

In patients with acute respiratory distress syndrome, mechanical ventilation often leads to ventilation-induced lung injury (VILI), which is attributed to unphysiological lung strain (UPLS) in respiratory dynamics. Platelet endothelial cell adhesion molecule-1 (PECAM-1), a transmembrane receptor, senses mechanical signals. The Src/STAT3 pathway plays a crucial role in the mechanotransduction network, concurrently triggering pyroptosis related inflammatory responses. We hypothesized that the mechanical stretch caused by UPLS can be sensed by PECAM-1 in the lungs, leading to VILI via the Src/STAT3 and pyroptosis pathway.

**Methods:**

A VILI model was established in rats through UPLS. The link between lung strain and VILI as well as the change in the activation of PECAM-1, Src/STAT3, and pyroptosis was firstly being explored. Then, the inhibitors of PECAM-1, Src, STAT3 were adopted respectively, the effect on VILI, inflammation, the Src/STAT3 pathway, and pyroptosis was evaluated. *In vitro*, human umbilical vein endothelial cells (HUVECs) were used to validate the findings *in vivo*.

**Results:**

UPLS activated PECAM-1, Src/STAT3 signaling pathway, inflammation, and pyroptosis in the VILI model with rats, whereas inhibition of PECAM-1 or the Src/STAT3 signaling pathway decreased lung injury, inflammatory responses, and pyroptosis. Inhibition of PECAM-1 also reduced activation of the Src/STAT3 signaling pathway. The mechanism was validated with HUVECs exposed to overload mechanical cyclic stretch.

**Conclusions:**

This study suggests that UPLS contributes to VILI by activating the PECAM-1/Src/STAT3 pathway and inducing inflammatory responses as well aspyroptosis.

## 1 Introduction

Acute respiratory distress syndrome (ARDS) is caused by intrapulmonary or extrapulmonary factors and causes a poor prognosis, particularly in older patients (Fang et al., 2020). ARDS treatment remains primarily supportive with lung protective ventilation (Pham and Rubenfeld, 2017; Bos and Ware, 2022; Gorman et al., 2022). However, mechanical ventilation (MV), as an artificial method, can trigger lung injury, termed ventilation-induced lung injury (VILI) (Slutsky and Ranieri, 2013; Nieman et al., 2022),with susceptibility increasing with aging (Setzer et al., 2013; Nin et al., 2008). Possible etiologies of VILI include volutrauma, atelectrauma, barotrauma, and biotrauma (Slutsky and Ranieri, 2013; Zochios et al., 2022). The overload of mechanical forces is sensed by mechanical signal receptors, which then activates relevant signal transduction pathways, induces the release of inflammatory factors, and finally converts harmful mechanical forces into biotrauma (Curley et al., 2016; Juffermans et al., 2022).

Mechanical forces during MV bring about stretch (stress) and deformation (strain) of lung tissue (Juffermans et al., 2022; Telias et al., 2022). In respiratory mechanics, lung stress refers to the force per unit area and is expressed in units of pressure, whereas lung strain refers to changes in lung volume caused by lung stress. According to the respiratory kinetic theory, lung strain is the ratio of the change in lung volume during respiration to the resting lung volume (△V/V), where V is the functional residual capacity (FRC) at 0cmH_2_O end-expiratory pressure (Gattinoni et al., 2012; Marini et al., 2020). Patients with “baby lung” caused by ARDS have a small FRC; therefore, a tidal volume (Vt) of 6–8 mL/kg of ideal body weight (IBW) might exceed the physiological limit of lung strain and induce VILI (González-López et al., 2012; Pelosi et al., 2021). For a patient, we called Patient A, if the FRC was 300 mL and the Vt was 450 mL, then the lung strain would be 1.5 (450 mL/300 mL), for another patient (called Patient B) with same IBW, but the FRC was 500 mL because of relative mild disease and the lung strain would be 0.9 (450 mL/500 mL) although the calculated Vt based on IBW would also be 450 mL, the same as Patient A. In other words, the development of VILI ultimately depends on whether the MV exceeds the physiological lung strain and stress, not only depends on the Vt based on IBW (Telias et al., 2022; Xie et al., 2017). Alternatively, excessive mechanical stress and strain on the lungs, rather than the ventilator itself, result in inflammatory responses in the lungs and cause VILI (Juffermans et al., 2022; Bai et al., 2022). A study on healthy pigs found that VILI developed when a lung strain was greater than the safe physiological threshold (1.5–2.0) (Protti et al., 2011). A lung strain that does not exceed the safe threshold is considered a physiological lung strain; otherwise, we call it an unphysiological lung strain (UPLS). The mechanism by which UPLS causes VILI remains debated. Therefore, exploring the pathological mechanisms of VILI is necessary to develop efficient prophylactic and therapeutic approaches for VILI during MV.

It is well known that VILI is caused by mechanical force, as a result, at the cellular level, the sensation and transmission of mechanical forces are key elements in the occurrence of VILI. When endothelial cells are subjected to mechanical forces, these forces are subsequently transmitted through cytoskeletal elements to cell-cell adhesion sites. Platelet endothelial cell adhesion molecule-1 (PECAM-1), an important component of these adhesion junctions among endothelial cells, directly transmits mechanical force, and comprises a mechanosensory complex with vascular endothelial-cadherin and vascular endothelial growth factor receptor 2 (Tzima et al., 2005). PECAM-1 senses and transmits the mechanical stretch and responds by activating downstream signaling pathways (Villar et al., 2019; Yamashiro and Yanagisawa, 2020; Givens and Tzima, 2016). One of these downstream mechanisms that has been previously confirmed is the Src/signal transducer and activator of transcription 3 (STAT3) pathway (McCormick et al., 2011; Privratsky and Newman, 2014).

There is a link between Src/STAT3 and pyroptosis (Le et al., 2022; Wei et al., 2023), which is a type of programmed cell death. Cellular pyroptosis and its associated inflammatory caspases play an essential role in the pathophysiology of various acute lung injuries (Lin et al., 2024; Ding et al., 2023; Shao et al., 2022). Phosphorylated Src can activate pyrin domain–containing-3 (NLRP3) inflammatory vesicles, which in turn activate polymorphonuclear neutrophils and exacerbate transfusion-related acute lung injury (Ding et al., 2023). However, whether the pyroptosis and VILI caused by UPLS, a mechanical stimulus, are mediated by PECAM-1 and Src/STAT3 pathway still needs to be elucidated. The regulatory relationship was investigated in this study with Wistar rats *in vivo* and human umbilical vein endothelial cells (HUVECs) *in vitro*.

## 2 Materials and methods

In this study, all animal protocols were approved by the Animal Experimentation Center’s Ethics Committee at Zhengzhou University (Ethical Review Number: ZZU-LAC20210305[3]). All animal experiments were conducted in accordance with the ARRIVE guidelines and the Guide for the Care and Use of Laboratory Animals by the National Research Council.

### 2.1 Reagents and animals

The reagents and assay kits adopted in this study were purchased from the commonly used qualified companies, and the information in detail was reported in Supplementary Table S1. Eighty-seven male Wistar rats (300–350 g, 7–8 weeks old) were purchased from Spelford Biotechnology Ltd. (Beijing, China),and three male C57BL/6 mice (20–25 g, 7–8 weeks old) were purchased from Changsheng Bio-technology (Liaoning, China). Animals were housed at a temperature between 22°C and 26°C with relative humidity between 40% and 60% and were provided with standard chow as well as sterile water. All animal tests were performed according to national and international ethical standards. Weight- and age-matched controls were used in all experiments. Only male Wistar rats were utilized in this study to reduce the potential impact of sex, a common practice that aligns with most prior experimental investigations, despite the lack of evidence regarding the influence of sex on the response to MV.

### 2.2 Study design

Three experiments were performed in this study to validate the hypothesis.

Experiment 1 was conducted to investigate the pathological characteristics, the activation of PECAM-1, Src/STAT3, and pyroptosis in the lungs by UPLS and in HUVECs by overload stretch. Twenty-four rats were randomly assigned to four groups: (Fang et al., 2020): Sham group (n = 6): animals received Sham operation without ventilation; (Pham and Rubenfeld, 2017); S1.0 group (n = 6): animals ventilated at a lung strain value of 1.0 for 4 h; (Bos and Ware, 2022); S1.5 group (n = 6): animals ventilated at a lung strain value of 1.5 for 4 h; and (Gorman et al., 2022) S2.0 group (n = 6): animals ventilated at a lung strain value of 2.0 for 4 h. *In vitro*, HUVECs were purchased from Kui Sai Biotechnology and cultured with complete culture medium (Sciencell,San Diego, CA, United States) in a 5% CO_2_ humidified and 37°C incubator. Cells were cultured into 3 ∼ 10 passages and seeded in a stretch chamber at 2 × 10^5^/cm^2^. After 24 h, the fresh medium was replaced and the cells were placed in the Shellpa Pro cell traction system (Menicon Life Science, Nagoya, Japanese) exposing to 5%, 15%, and 20% cyclic stretch (CS) for 30 times/min for 4 h (sine wave, 1s deformation and 1s relaxation alternately). Cell culture supernatant was analyzed using lactate dehydrogenase (LDH) cytotoxicity assay kit to assess the severity of cell injury.

Experiment 2 was conducted to determine the effect of blocking PECAM-1 on VILI, the downstream Src/STAT3 pathway, and pyroptosis with *in vivo* and *in vitro* experiments. The PECAM-1 Ab (MEC13.3) used in this study was purchased from Bio Legend (San Diego, United States) and the catalog is 102530. The immunogen of PECAM-1 Ab is Polyoma middle T transformed EC line tEnd.1., and the isotype is Rat IgG2a, κ. The reactivity of PECAM-1 Ab to rat lung tissue was verified by Western blot (WB) with PECAM-1 Ab (1:500 dilution) as the primary antibody in healthy male Wistar rats and compared with C57BL/6 mice. Endothelial cells were only one of the components in lung tissues and the content of PECAM-1 was relatively low in the intact lung tissue, therefore, CO- immunoprecipitation was employed to enrich and purify PECAM-1 protein. For HUVECs, PECAM-1 was directly tested by WB to confirm the specific binding of PECAM-1 Ab to the immunogen of PECAM-1. Twenty-four rats were randomly assigned to four groups: (Fang et al., 2020): Sham group (n = 6); (Pham and Rubenfeld, 2017); MV group (n = 6); (Bos and Ware, 2022); MV + anti-PECAM-1 antibody (PECAM-1 Ab) group (n = 6): animals treated with PECAM-1 Ab and received MV for 4 h; and (Gorman et al., 2022) MV + Rabbit IgG group (n = 6): animals treated with Rabbit IgG and received MV for 4 h (the Rabbit IgG as control). The Vt was calculated to achieve a lung strain value of 2.0. The dose of PECAM-1 Ab (5 mg/kg body weight, tail vein injection) was referred from one previous study (Winneberger et al., 2021) and the dose of Rabbit IgG was 5 mg/kg body weight (tail vein injection) (Gumina et al., 1996).

*In vitro*, concentration for 50% of maximal effect (EC50) of PECAM-1 Ab was confirmed through cell viability measured by Cell Counting Kit-8 (CCK-8). HUVECs were cultured in 96-well plates (100 μL/well) at 1 × 10^4^/cm^2^. After 24 h, PECAM-1 Ab was incubated at concentrations of 0.01, 0.1, 1, 10, 100 ng/μL for 24 h, respectively. 10 μL of CCK-8 solution was added to each well, and absorbance at 450 nm was detected with a microplate reader (Molecular devices, Shanghai, China) after incubation for 4 h. Nonlinear regression analysis was employed to model the relationship between cell viability and corresponding concentrations. The concentration that elicits 50% of the maximum cell viability is referred to as the EC50 of PECAM-1 Ab. The PECAM-1 Ab or Rabbit IgG was administered for interventional groups 30 min prior to CS (15% elongation). The HUVECs in stretching control group only received CS (15% elongation) and the sham group was also cultured in the chamber using the same incubator.

Experiment 3 was conducted to determine the effect of Src/STAT3 inhibition on VILI, the activation of PECAM-1 and pyroptosis in the lungs. Thirty-six animals were randomly assigned into six groups: (Fang et al., 2020): Sham group (n = 6); (Pham and Rubenfeld, 2017); Sham + SU6656 (Src inhibitor) group (n = 6): animals treated with SU6656 and received Sham operation; (Bos and Ware, 2022); Sham + BP-1-102 group (n = 6): animals treated with BP-1-102 and received Sham operation; (Gorman et al., 2022); MV group (n = 6): animals ventilated for 4 h; (Slutsky and Ranieri, 2013); MV + SU6656 group (n = 6): animals treated with SU6656 and received MV for 4 h; and (Nieman et al., 2022) MV + BP-1-102 group (n = 6): animals treated with BP-1-102 and received MV for 4 h. Vt was calculated by lung strain value of 2.0 Vt was calculated to achieve a lung strain value of 2.0.

### 2.3 VILI model developed by UPLS and the interventions

Prior to tracheal intubation, the rats were fasted, including from water, for 6 h, and were subsequently anesthetized via intraperitoneal injection of sodium pentobarbital (40 mg/kg body weight, final concentration 2% in saline). One-third of the initial dose was injected every 1.5 h to maintain the level of anesthesia. A sterile 14G cannula was used for tracheal intubation. The catheter was placed in the trachea and attached to an animal ventilator (Alcott Biotech, Shanghai, China) for MV. The animal ventilator was calibrated as follows: Vt was calculated by the lung strain value from the FRC and was maintained unchanged during the experiment, given that UPLS is considered as lung strain up to 1.5 based on the previous studies (Xie et al., 2017); the respiratory rate was 60 breaths/min; inspiratory to expiratory ratio was 1:1; PEEP was 0 cmH2O; oxygen concentration was 21%; and the duration of MV was 4 h. The FRC was calculated based on body weight using the reported formula (Yokoyama, 1983). The details of weight, FRC, and Vt are reported in Supplementary Table S2. In some experiments, rats were treated with SU6656 (8 mg/kg, diluted in 5% dimethylsulfoxide [DMSO], intraperitoneal injection), STAT3 inhibitor BP-1-102 (10 mg/kg, diluted in 5% DMSO, intraperitoneal injection), PECAM-1 Ab (5 mg/kg, final concentration 10% in saline, tail vein injection), or Rabbit IgG (5 mg/kg, final concentration 10% in saline, tail vein injection) 30 min before tracheal intubation (Ward et al., 1993; Severgnini et al., 2005; Xu et al., 2020). After 4 h of MV, all rats were administered sodium pentobarbital solution (80 mg/kg) intraperitoneally and sacrificed, and the abdominal aorta was cut.

### 2.4 Lung wet/dry (W/D) weight ratio and lung injure scoring

After 4 h of MV, the surface of the right upper lobe was dried with gauze, and the right upper lobes were measured using an analytical balance to determine the wet weight. Next, the lungs were placed in an oven at 60°C to dry for 24 h, after which the dry weight was determined. The wet weight was divided by the dry weight to obtain the W/D weight ratio. The lung injure scoring system was referenced the previous study (Lin et al., 2018). The middle lobe of the right lung was placed into a container filled with paraformaldehyde. Negative pressure was achieved through pumping, which expelled gas from the lung tissue and allowed paraformaldehyde to enter. This process involved decompression every 6 h and lasted for 24 h to ensure fixation. Lung slices were fixed in paraffin and stained with hematoxylin and eosin (HE) to detect pathological alterations in each tissue set. The severity of lung injury was evaluated based on four main pathological changes such as neutrophils’ infiltration, hemorrhages, alveolar congestion and thickening of the alveolar walls. Each pathological change was awarded up to four scores: minimal or negligible injury 0; mild injury 1; moderate injury 2; serious injury 3; and maximal injury 4. The sum of the scores for the four pathological changes was calculated for each field of view. For each animal, three non-overlapping fields of view are taken for pathological scoring, and the average is calculated. Two pathologists, blinded to the experimental treatment, examined all the tissue sections under a light microscope.

### 2.5 Protein concentrations and cell counts in bronchoalveolar lavage fluid (BALF)

The left lung was lavaged with 3 mL of pre-cooled PBS via tracheal intubation, and the fluid was removed after 10 s. This process was repeated three times, resulting in recovery rates exceeding 85%. After centrifugation of the BALF, the protein concentration in the supernatant, an indicator of pulmonary permeability, was determined using a BCA assay kit. The pelleted cells which had settled at the bottom were stained with Wright-Giemsa stain and then classified and counted under a microscope (Nikon, Eclipse Ni-U, Tokyo, Japan) to assess the inflammatory infiltration of neutrophils and macrophages.

### 2.6 Enzyme-linked immunosorbent assay (ELISA)

The levels of TNF-α and IL-1β in BALF and serum were measured using the Rat TNF-α and IL-1β ELISA kits, respectively. Similarly, the levels of vWF and ET-1 in serum were measured using the Rat vWF and ET-1 ELISA kit, respectively. All experimental procedures were conducted according to the manufacturer’s instructions.

### 2.7 Immunohistochemistry

Paraffin-embedded lung tissue slices were analyzed immunohistochemically for CD11b and MPO simultaneously to accurately detect neutrophil activation (Liu et al., 2022).

### 2.8 Immunofluorescence (IF)

Paraffin-embedded lung tissue sections were incubated overnight at 4°C with anti-CD11b and anti-MPO. Next, samples were washed three times with PBS and incubated with Cy3 and Alexa Fluor 488-conjugated species-specific secondary antibodies. The nuclei were subsequently counterstained with DAPI (4′,6-diamidino-2-phenylindole) to identify the nuclei. Finally, sections were observed under a fluorescence microscope. Images were magnified at ×400 for quantitative analysis using ImageJ software.

### 2.9 Protein extraction and WB

Lung tissue was homogenized at 4°C in RIPA buffer to obtain total protein. Protein concentrations were determined using a BCA kit. Protein samples were loaded, electrophoresed on sodium dodecyl sulfate-polyacrylamide gels, and transferred to polyvinylidene fluoride membranes. The membranes were blocked with Fast Blocking Solution for 20 min at room temperature (RT, 22°C–23°C) and incubated overnight at 4°C with the following primary antibodies: anti-Src (1:1,000), anti-p-Src Y419 (1:1,000), anti-p-Src Y527 (1:1,000), anti-STAT3 S727 (1:1,000), anti-p-STAT3 Y705 (1:1,000), anti-STAT3 (1:1,000), anti-PECAM-1 (1:1000), anti-p-PECAM-1 (1:1,000), anti-NLRP3 (1:1,000), anti-ASC (1:1,000), anti-Caspase-1 (1:1,000), anti-IL-1β (1:1,000), anti- gasdermin D (GSDMD)-N (1:500), and anti-β-actin (1:2,000). The membranes were incubated the following day for 1 h at RT with anti-rabbit or anti-mouse IgG secondary antibodies (1:5,000) coupled with HRP. Finally, the membranes were imaged using an AI680 ultrasensitive multifunctional imager with enhanced chemiluminescence. ImageJ software was used to perform semi-quantitative analysis.

### 2.10 Co-immunoprecipitation

Protein G magnetic beads were incubated with PECAM-1 Ab (5 μg/mL) overnight at 4°C for antibody adsorption. Lung tissue protein supernatant was incubated with magnetic beads bound to the antibody overnight at 4°C for immunoprecipitation, and after the supernatant was removed, SDS-PAGE Sample Loading Buffer (1X) was added and heated at 95°C for 5 min. Place on a magnetic stand for 10 s and remove the supernatant for Western detection.

### 2.11 Statistical analysis

Data are presented as mean ± standard deviation (SD). Normality was assessed using the Shapiro-Wilk method, and homogeneity of variances was tested using the Levene test. The overall difference in means among multiple groups was tested using one-way analysis of variance (ANOVA) for the data with normal distribution and homogeneity of variances, and further pairwise comparisons were conducted using the Student-Newman-Keuls test for the outcomes showing significant differences in the overall population by one-way ANOVA, otherwise, Kruskal-Wallis H and Dunn’s tests were adopted. All tests were two-sided, and *p* < 0.05 was considered statistically significant. Statistical analyses were conducted using SPSS (version 27.0, IBM, Armonk, NY, United States).

## 3 Results

### 3.1 Association between lung strain and VILI, as well as the potential changes in PECAM-1, Src/STAT3 and pyroptosis

Experiment 1 was conducted to evaluate the correlation between VILI and UPLS as well as the potential changes in PECAM-1, Src/STAT3 and Pyroptosis with *in vivo* and *in vitro* experiments.

#### 3.1.1 UPLS induces lung injury

**FIGURE 1:**
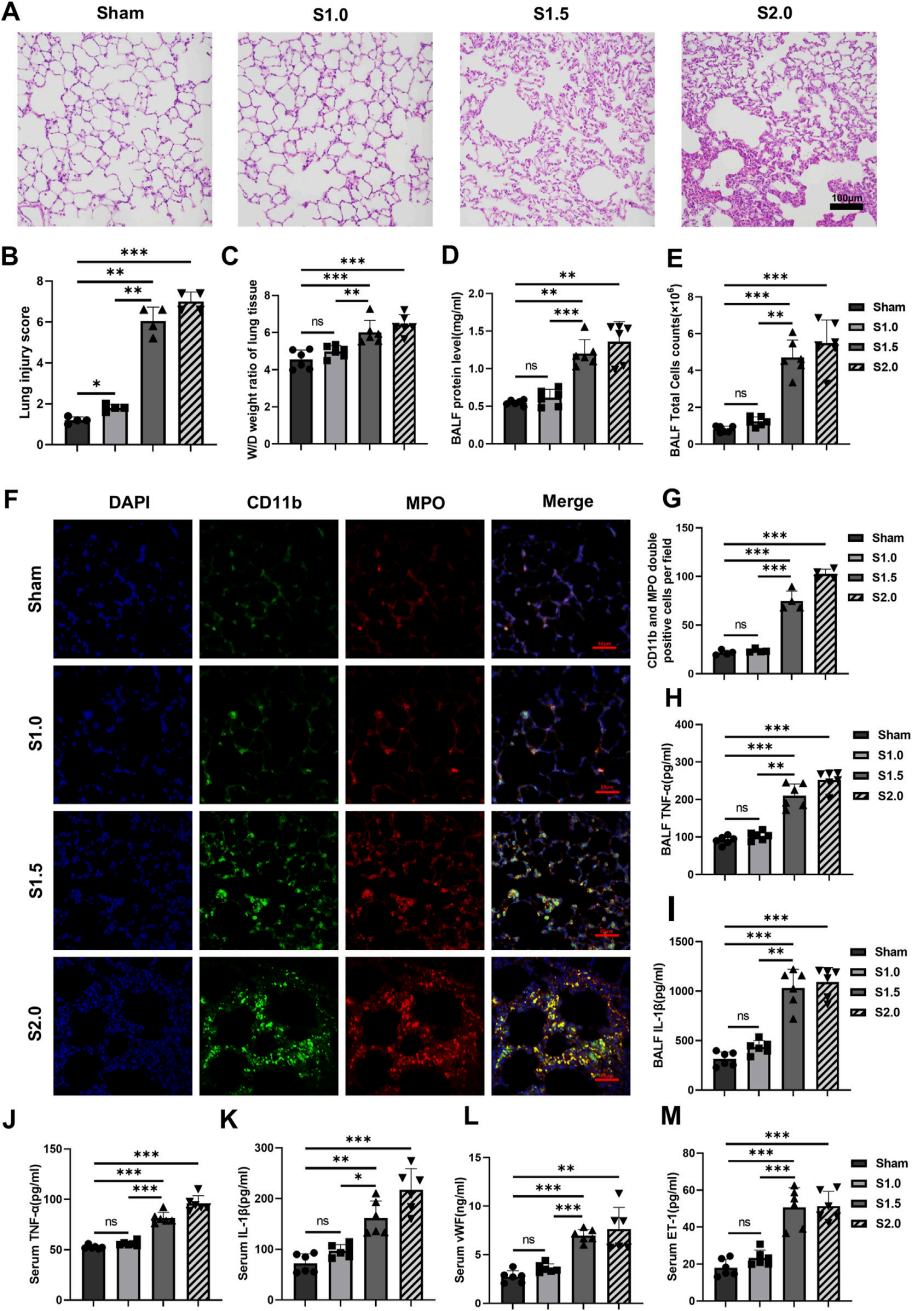
Increased lung strain exacerbates lung injury and inflammatory response **(A)** Histopathological analysis (scale: 100 μm, magnification ×200). **(B)** Lung injury score (n = 4, F = 189.091, *p* < 0.001). **(C)** W/D weight ratio of rat lungs (n = 6, F = 17.224, *p* < 0.001). **(D)** Total protein concentration (F = 33.648, *p* < 0.001) and **(E)** total cell counts (F = 52.534, *p* < 0.001) in BALF (n = 6). **(F)** Representative images of IF staining of CD11b (green) and MPO (red) of different groups (scale: 50 μm, magnification ×400, n = 4). **(G)** Quantitative analysis of CD11b and MPO double-positive cells (F = 180.928, *p* < 0.001) in IF images. **(H, I)** TNF-α (F = 79.220, *p* < 0.001) and IL-1β (F = 57.262, *p* < 0.001) levels in BALF (n = 6). **(J–M)** TNF-α (F = 103.538, *p* < 0.001), IL-1β (F = 30.325, *p* < 0.001), vWF (F = 23.310, *p* < 0.001), and ET-1 (F = 34.801, *p* < 0.001) levels in serum (n = 6). Data are shown as mean ± SD; *p < 0.05, **p < 0.01, ***p < 0.001, ns no significance. W/D: Wet/Dry; BALF: bronchoalveolar lavage fluid; MPO: myeloperoxidase; IF: immunofluorescence; TNF: tumor necrosis factor; vWf: von Willebrand factor; IL: interleukin; ET-1: endothelin-1; SD: standard deviation.

HE stains of lung tissue was performed to evaluate for lung injury in VILI models with rats. The Sham and S1.0 groups demonstrated no or minor pathological injuries. Significant numbers of inflammatory cell infiltrated, destroyed alveolar structures, and hemorrhages were observed in the lung tissues of the S1.5 and S2.0 groups (Figure 1A). The degree of lung injury scores markedly increased when the lung strain reached 1.5 (Figure 1B). The lung W/D ratios, BALF protein concentrations, and BALF cell counts in S1.5 and S2.0 groups were higher than those in the Sham and S1.0 groups (Figures 1C–E) with similar concurrent changes in BALF neutrophils and macrophages count (Supplementary Figures S1A, B).

#### 3.1.2 UPLS induces inflammatory responses in the lung

IF staining of lung tissues observed under a 400× fluorescent microscope showed few MPO and CD11b double-positive cells in the lung tissues of the Sham and S1.0 groups. In contrast, the S1.5 and S2.0 groups showed a significant increase in MPO and CD11b double-positive cells in their lung tissues compared to the Sham group (Figures 1F, G). Meanwhile, immunohistochemistry staining and quantitative analysis showed that the expression levels of CD11b and MPO were significantly higher in the lung tissues of the S 1.5 and S2.0 groups (Supplementary Figures S1C–E), these changes suggested increased inflammatory cell infiltration. The levels of TNF-α and IL-1β in BALF, and TNF-α, IL-1β, vWF, and ET-1 in serum increased significant in S1.5 group and S2.0 group compared to the Sham group. No significant differences of these biomarkers were observed between the S1.0 group and Sham group (Figures 1H–M). These findings indicated that the lung inflammatory response was significantly aggravated when lung strain was increased from 1.0 to 1.5.

#### 3.1.3 UPLS activates PECAM-1, the Src/STAT3 signaling pathway, and pyroptosis

**FIGURE 2:**
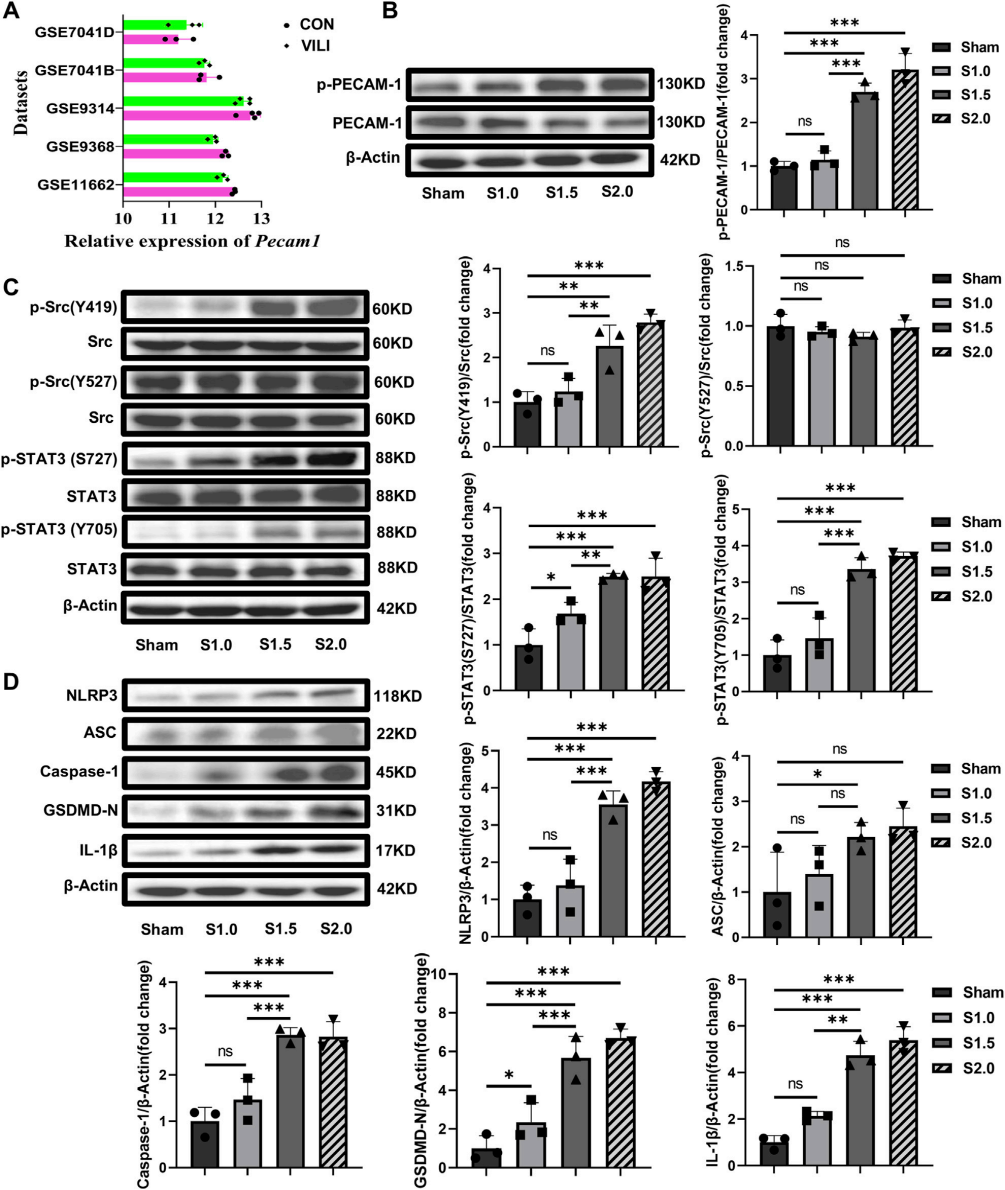
Increased lung strain Activates PECAM-1, the Src/STAT3 Signaling Pathway, and Pyroptosis **(A)**The databases (GSE7041, GSE9314, GSE9368, GSE11662) were searched for relevant gene, the level of mRNA of PECAM-1 in the VILI group mice was not higher than that in the control group.**(B)** WB analysis of p-PECAM-1 and PECAM-1 in lung tissue with different lung strain (F = 64.174, *p* < 0.001). **(C)** WB analysis of p-Src, Src, p-STAT3, and STAT3 in lung tissue, p-Src (Y419)/Src (F = 21.281, *p* < 0.001), p-Src (Y527)/Src (F = 1.080, *p* = 0.411), p-STAT3 (S727)/Src (F = 18.109, *p* < 0.001), p-STAT3 (Y705)/Src (F = 36.863, *p* < 0.001). **(D)** WB analysis of pyroptosis-related proteins in lung tissue, NLRP3 (F = 35.094, *p* < 0.001), ASC (F = 3.882, *p* = 0.055), Caspase-1 (F = 25.260, *p* < 0.001), GSDMD-N (F = 30.486, *p* < 0.001), IL-1β (F = 63.954, *p* < 0.001). Data are shown as mean ± SD (n = 3). *p < 0.05, **p < 0.01, ***p < 0.001, ns no significance. PECAM-1: platelet endothelial cell adhesion molecule-1; STAT3: signal transducer and activator of transcription 3; WB: Western blot.

WB was used to detect the activation of PECAM-1, the Src/STAT3 signaling pathway, and pyroptosis to determine their involvement in the pathophysiology of VILI. Before the experiments, we searched relevant gene databases (GSE7041, GSE9314, GSE9368, GSE11662) and found that the level of mRNA of PECAM-1 in the VILI group mice was not higher than that in the control group (Figure 2A). Therefore, we considered that PECAM-1 might be exerting its effects through other mechanisms, such as phosphorylation. We found the levels of pPECAM-1, pSrc Y419, pSTAT3 S727, and pSTAT3 Y705 were significantly elevated in the S1.5 and S2.0 groups compared with the Sham group whereas the level of pSrc Y527 was not significantly changed (Figures 2B, C) *in vivo* experiments with Rats. Furthermore, pyroptosis-related protein expressions were significantly elevated in the S1.5 and S2.0 groups compared with the Sham group (Figure 2D). These results demonstrated that PECAM-1 and the Src/STAT3 signaling pathway were activated, and pyroptosis increased as the lung strain reached 1.5.

**FIGURE 3:**
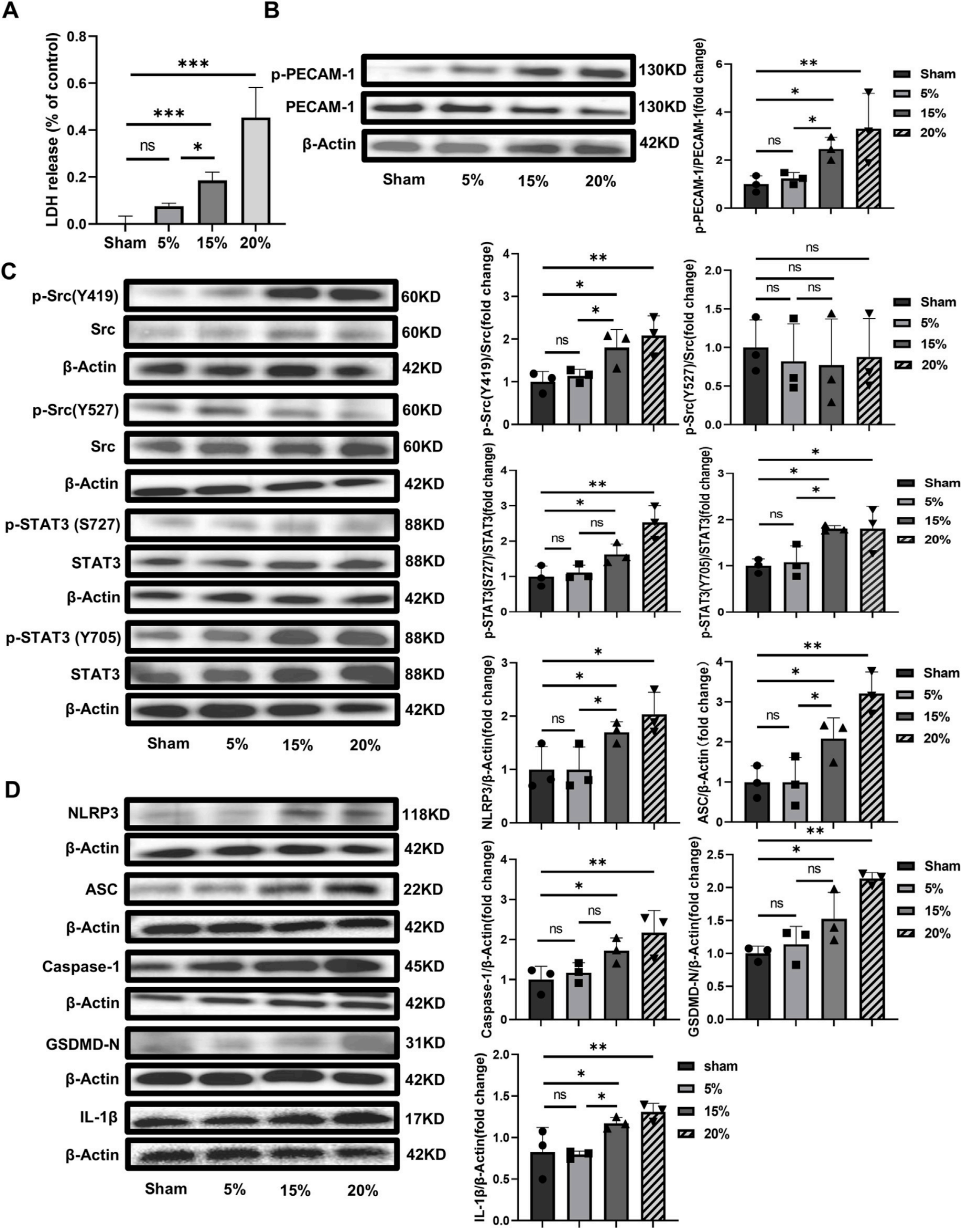
Cell damage and the potential mechanism induced by CS *in vitro* with HUVECs **(A)** CS with 15% or 20% elongation resulted in increased LDH release (F = 50.040, *p* < 0.001); **(B)** WB analysis of PECAM-1 (phosphorylated and unphosphorylated) in HUVECs (F = 5.455, *p* = 0.02); **(C)** WB analysis of Src (phosphorylated and unphosphorylated) and STAT3 (phosphorylated and unphosphorylated). p-Src (Y419)/Src (F = 6.780, *p* = 0.014), p-Src (Y527)/Src (F = 0.119, *p* = 0.946), p-STAT3(S727)/Src (F = 13.164, *p* = 0.002), **(D)** WB analysis of the related proteins to pyroptosis including NLRP3, ASC, Caspase-1, GSDMD-N and IL-1β, NLRP3 (F = 5.642, *p* = 0.023), ASC (F = 12.265, *p* = 0.002), Caspase-1 (F = 5.821, *p* = 0.021), GSDMD-N (F = 12.063, *p* = 0.002), IL-1β (F = 7.373, *p* = 0.011). Data are shown as mean ± SD (n = 3). *p < 0.05, **p < 0.01, ns no significance. PECAM-1: platelet endothelial cell adhesion molecule-1; STAT3: signal transducer and activator of transcription 3; WB: Western blot; SD: standard deviation.

*In vitro* experiments with HUVECs, CS with 15% or 20% elongation resulted in increased LDH release and meant significant cell damage (Figure 3A). The levels of pPECAM-1 (Figure 3B), pSrc Y419, pSTAT3 S727, and pSTAT3 Y705 (Figure 3C) were higher in the groups exposed to overload CS whereas the difference in the levels of pSrc Y527 was not significant. Additionally, the levels of NLRP3, ASC, Caspase-1, GSDMD-N and IL-1β were significantly elevated in the overload CS groups compared with the Sham group (Figure 3D).

### 3.2 PECAM-1 Ab alleviates VILI, the downstream Src/STAT3 pathway and pyroptosis

In experiment 1, we found pPECAM-1, pSrc Y419, pSTAT3 S727, and pSTAT3 Y705 were significantly elevated. Studies *in vitro* (Muller et al., 1993) and *in vivo* (Winneberger et al., 2021) have shown that the function of PECAM-1 could be blocked by its specific antibody. The objective of experiment 2 was to examine the effects of PECAM-1 Ab on VILI, the Src/STAT3 signaling pathway activation, and pyroptosis *in vivo* and validated with *in vitro* experiments. The reactivity of the PECAM-1 Ab was confirmed in Wistar rats (Supplementary Figure S2A) and HUVECs (Supplementary Figure S2B).

#### 3.2.1 PECAM-1 Ab relieves lung injury

**FIGURE 4:**
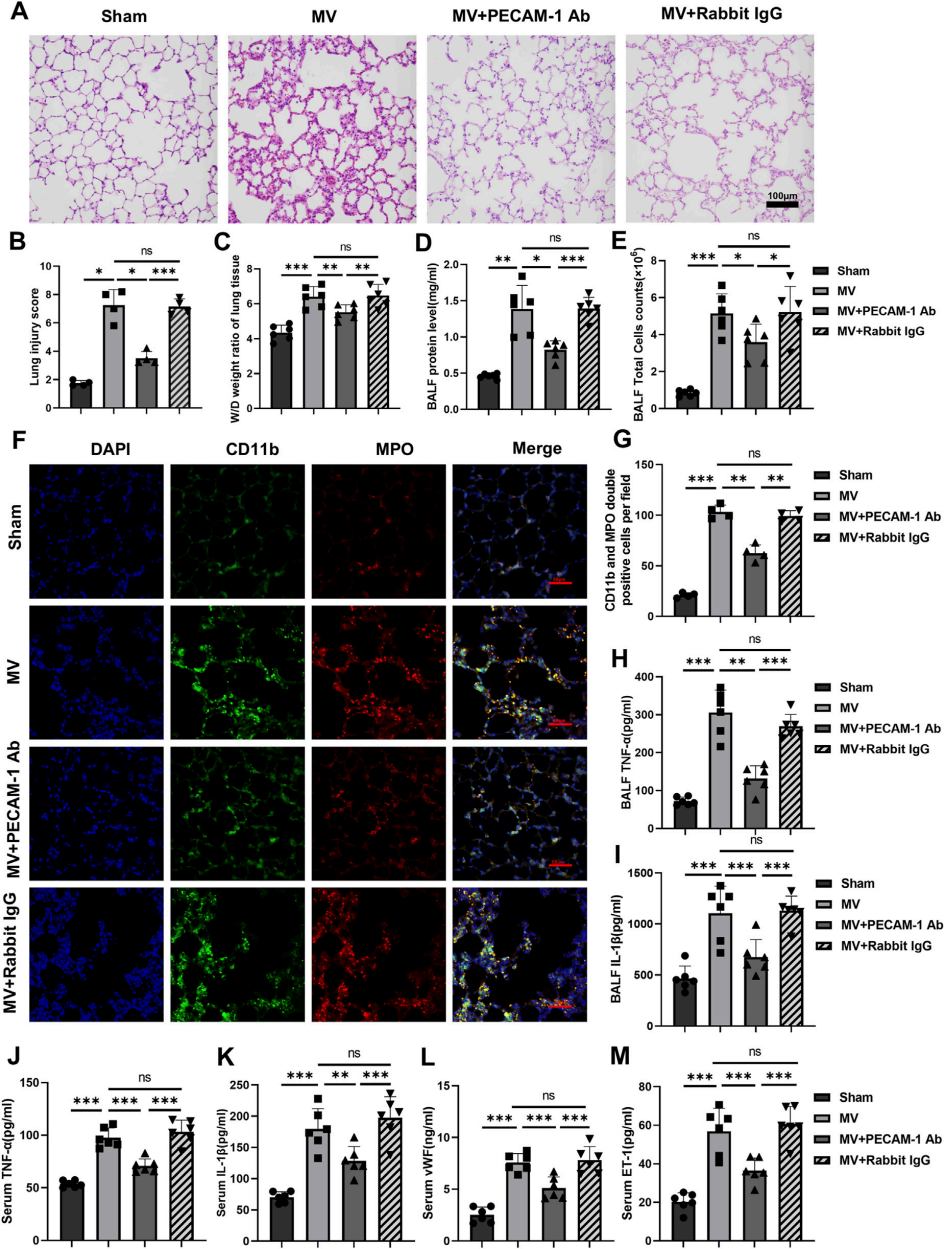
PECAM-1 Ab relieves lung injury and inflammation. **(A)** Histopathological analysis and **(B)** degree of lung injury (scale: 100 μm, magnification ×200, n = 4, F = 67.443, *p* < 0.001). **(C)** W/D weight ratio of rat lungs (n = 6, F = 21.170, *p* < 0.001). **(D)** Total protein concentration (F = 34.753, *p* < 0.001) and **(E)** total cells counts (F = 24.976, *p* < 0.001) in BALF (n = 6). **(F)** Representative images of IF staining of CD11b (green) and MPO (red) of different groups (scale: 50 μm, magnification ×400, n = 4). **(G)** Quantitative analysis of CD11b and MPO double-positive cells (F = 161.377, *p* < 0.001) in IF images. **(H, I)** TNF-α (F = 51.301, *p* < 0.001) and IL-1β (F = 18.901, *p* < 0.001) levels in BALF (n = 6). **(J–M)** TNF-α (F = 48.896, *p* < 0.001), IL-1β (F = 27.593, *p* < 0.001), vWF (F = 35.916, *p* < 0.001) and ET-1 (F = 29.462, *p* < 0.001) levels in serum (n = 6). Data are shown as mean ± SD; *p < 0.05, **p < 0.01, ***p < 0.001, ns no significance. PECAM-1 Ab: platelet endothelial cell adhesion molecule-1 antibody; W/D: Wet/Dry; BALF: bronchoalveolar lavage fluid; MPO: myeloperoxidase; IF: immunofluorescence; TNF: tumor necrosis factor; vWf: von Willebrand factor; IL: interleukin; ET-1: endothelin-1; SD: standard deviation.

Lung tissue injury and the pathological score for lung injury in the MV + PECAM-1 Ab group were significantly reduced compared to those in the MV group (Figures 4A, B). Moreover, the lung tissue W/D weight ratio, BALF protein concentration, and BALF cell count were significantly reduced in the MV + PECAM-1 Ab group compared to the MV group (Figures 4C–E; Supplementary Figures S3A, B).

#### 3.2.2 PECAM-1 Ab decreases lung inflammation

The number of MPO and CD11b double-positive cells in lung tissues was lower in the MV + PECAM-1 Ab group than in the MV group; however, no significant differences were observed between the MV + Rabbit IgG and MV groups by IF(Figures 4F, G). Meanwhile, the expression levels of MPO and CD11b in the lung tissues of the MV + PECAM-1 Ab group were lower than those in the MV group; however, MPO and CD11b in the lung tissues of the MV + Rabbit IgG group were not significantly different from those in the MV group through immunohistochemistry (Supplementary Figures S3C–E). The group that exposed to MV + PECAM-1 Ab showed a significant reduction in TNF-α and IL-1β levels in BALF, as well as TNF-α, IL-1β, vWF, and ET-1 levels in serum compared with the MV group (Figures 4H–M).

#### 3.2.3 PECAM-1 Ab inhibits the Src-STAT3 signaling pathway and pyroptosis

**FIGURE 5:**
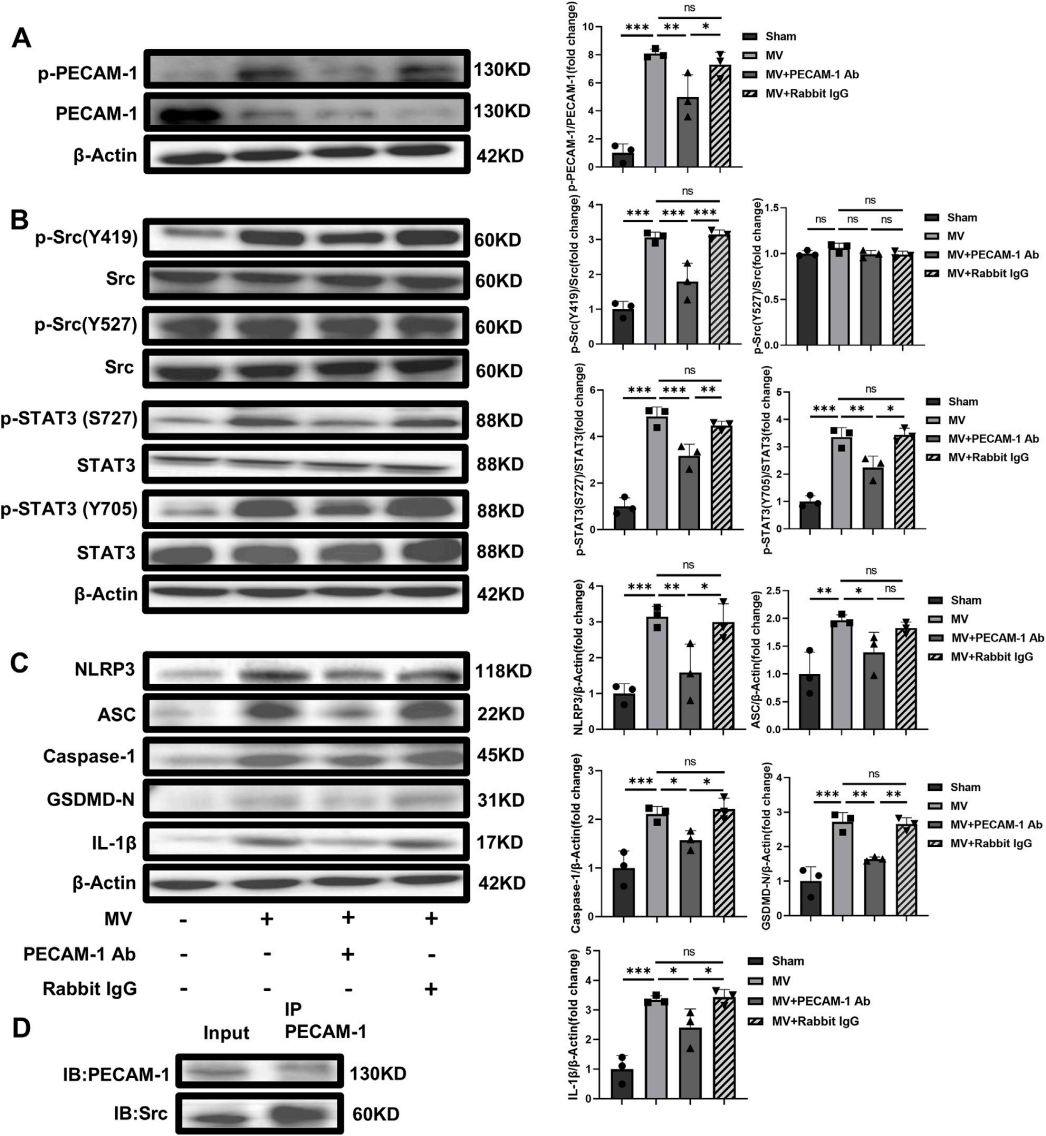
PECAM-1 Ab inhibits the Src-STAT3 signaling pathway and pyroptosis activation. **(A)** WB analysis of p-PECAM-1 and PECAM-1 in lung tissue (F = 31.418, *p* < 0.001). **(B)** WB analysis of p-Src, Src, p-STAT3, and STAT3 in lung tissue, p-Src (Y419)/Src (F = 35.283, *p* < 0.001), p-Src (Y527)/Src (F = 1.995, *p* = 0.193), p-STAT3 (S727)/Src (F = 61.182, *p* < 0.001), p-STAT3(Y705)/Src (F = 38.739, *p* < 0.001). **(C)** WB analysis of pyroptosis-related proteins in lung tissue, NLRP3 (F = 12.637, *p* = 0.002), ASC (F = 7.533, *p* = 0.01), Caspase-1 (F = 15.751, *p* = 0.001), GSDMD-N (F = 29.604, *p* < 0.001), IL-1β (F = 22.273, *p* < 0.001). **(D)** The interaction between Src and PECAM-1 by Co-Immunoprecipitation. Data are shown as mean ± SD (n = 3). *p < 0.05, **p < 0.01, ***p < 0.001, ns no significance. PECAM-1: platelet endothelial cell adhesion molecule-1; STAT3: signal transducer and activator of transcription 3; WB: Western blot; SD: standard deviation; Ab: antibody, IP: Immunoprecipitation; IB: Immunoblotting.

The levels of pPECAM-1, pSrc Y419, pSTAT3 S727, and pSTAT3 Y705 and pyroptosis-related proteins expression were reduced in the MV + PECAM-1 Ab group compared with the MV group. The levels of pPECAM-1, pSrc Y419, pSTAT3 S727, and pSTAT3 Y705 and pyroptosis-related protein expression in the MV + Rabbit IgG group did not significantly differ from those in the MV group. However, the levels of pSrc Y527 did not significantly change in the MV + PECAM-1 Ab and MV + Rabbit IgG groups compared with the MV group (Figures 5A–C). This suggests that PECAM-1 affects the Src/STAT3 signaling pathway through pSrc Y419, rather than pSrc Y527, and both phosphorylated sites of STAT3 (S727 and Y705). Co-immunoprecipitation found that Src was interacted with PECAM-1 (Figure 5D).

**FIGURE 6:**
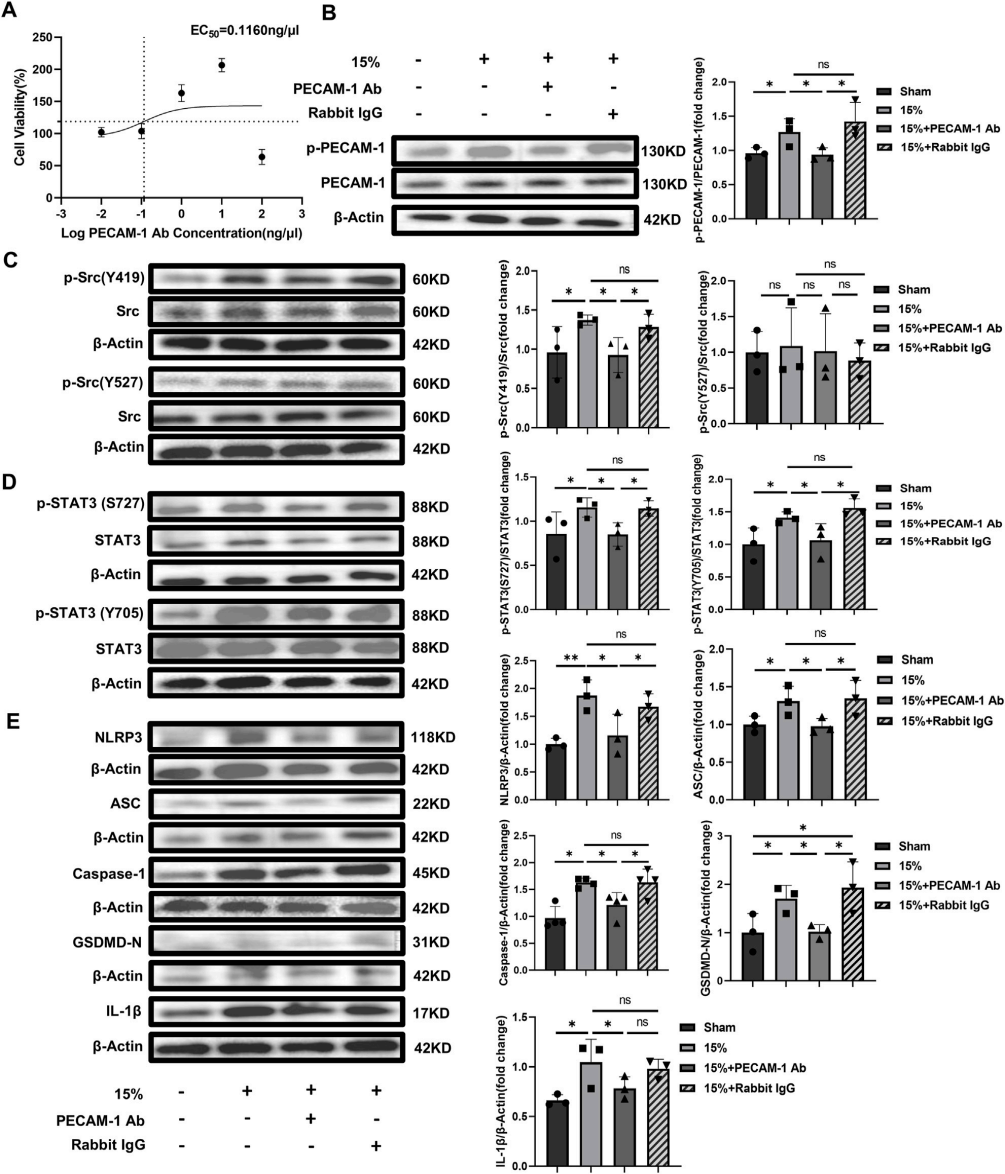
The effect of PECAM-1 Ab on signaling pathway and pyroptosis *in vitro* with HUVECs **(A)** Dose-response curve of PECAM-1 Ab. The horizontal dashed line represents 50% of the maximum effect, the vertical dashed line represents the PECAM-1 Ab concentration corresponding to 50% of the maximum effect, compared to the control, the baseline cell variability was 94.29% and the cell variability at maximum effect was 143.3%; **(B)** WB analysis of PECAM-1 (phosphorylated and unphosphorylated) in HUVECs (F = 5.454, *p* < 0.025); **(C)** WB analysis of Src (phosphorylated and unphosphorylated). p-Src (Y419)/Src (F = 3.262, *p* = 0.080), p-Src (Y527)/Src (F = 0.123, *p* = 0.944); **(D)** WB analysis of STAT3 (phosphorylated and unphosphorylated) p-STAT3 (S727)/Src (F = 3.577, *p* = 0.066), p-STAT3 (Y705)/Src (F = 5.272, *p* = 0.012); **(E)** WB analysis of the related proteins to pyroptosis including NLRP3 (F = 7.197, *p* = 0.002), ASC (F = 3.991, *p* = 0.052), Caspase-1 (F = 2.814, *p* = 0.108), GSDMD-N (F = 6.123, *p* = 0.018), and IL-1β(F = 4.761, *p* = 0.034). Data are shown as mean ± SD (n = 3). *p < 0.05, **p < 0.01, ns no significance. PECAM-1: platelet endothelial cell adhesion molecule-1; STAT3: signal transducer and activator of transcription 3; WB: Western blot; SD: standard deviation.

*In vitro* experiments with HUVECs, different PECAM-1 Ab concentrations (0.01, 0.1, 1, 10 and 100 ng/μL) were firstly used to explore the appropriate dosage according to cell viability by CCK-8. The effective dosage ranged from 0.01 ng/μL to 10 ng/μL, in which PECAM-1 Ab enhanced cell viability (Figure 6A) and the EC50 was 0.1160 ng/μL. The concentration used in these *in vitro* experiments was 10 ng/μL. The phosphorylation level of PECAM-1 was higher in the group exposed to overload CS (15% elongation) than that in the Sham, while the phosphorylation of PECAM-1 could be alleviated by the use of PECAM-1 Ab although the HUVECs were exposed to the same overload CS, which indicated that the application of PECAM-1 Ab had neutralized PECAM-1 on the cell membrane (Figure 6B). Additionally, the levels of pSrc Y419 (Figure 6C), pSTAT3 S727, pSTAT3 Y705 (Figure 6D), NLRP3, ASC, Caspase-1, GSDMD-N and IL-1β were increased by CS (15% elongation),and they could be offset by PECAM-1 Ab whereas could not be affected by non-specific rabbit IgG (Figure 6E).

### 3.3 Inhibitors of the Src/STAT3 pathway reduces VILI, without affecting PECAM-1

Experiment 3 examines whether Src/STAT3 is the downstream signaling pathway of PECAM-1 in VILI caused by UPLS.

#### 3.3.1 Inhibitors of Src/STAT3 pathway relieves the lung injury

**FIGURE 7:**
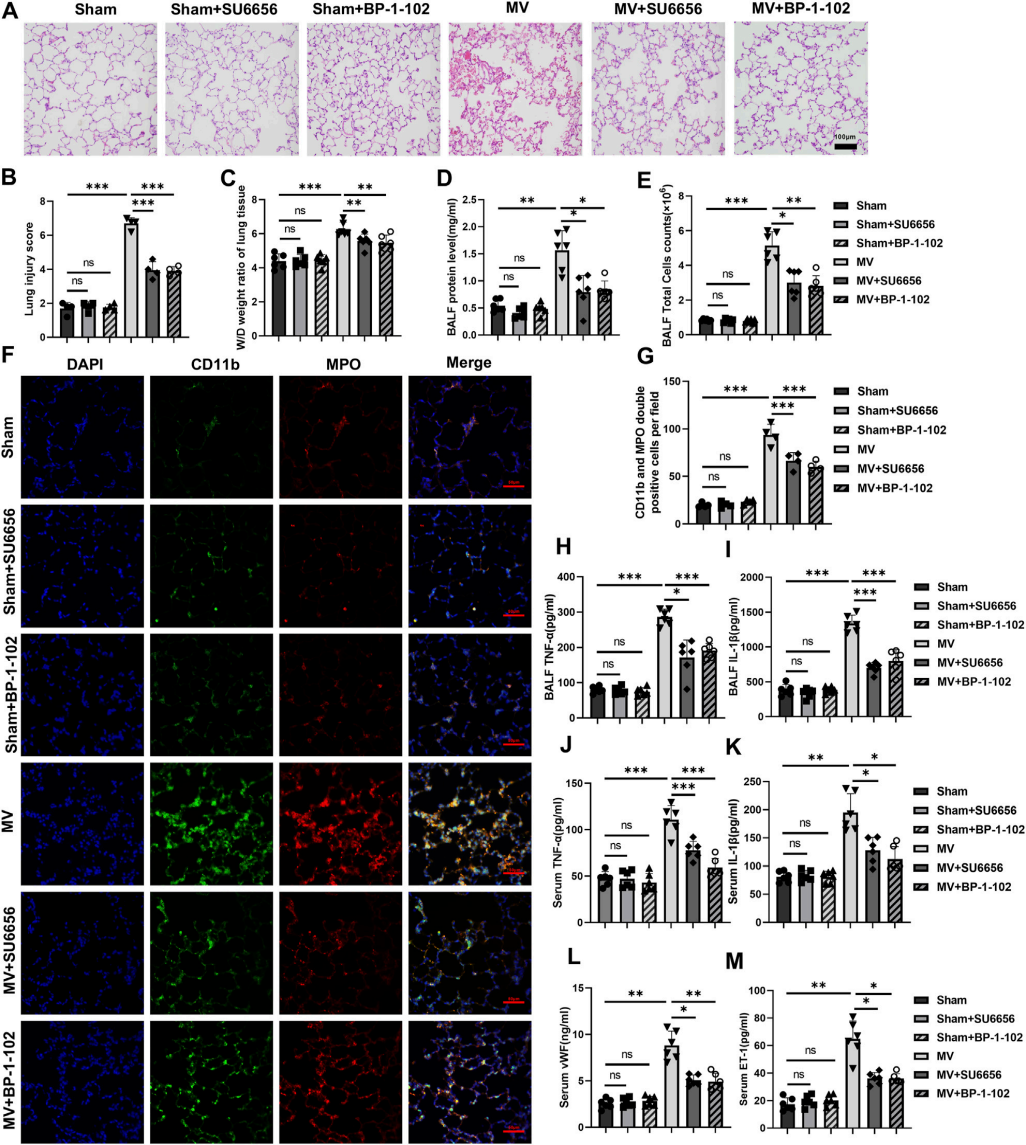
Downregulation of the Src/STAT3 signaling pathway reduces lung injury and inflammatory response **(A)** Histopathological analysis and **(B)** degree of lung injury (scale: 100 μm, magnification ×200, n = 4, F = 139.402, *p* < 0.001). **(C)** W/D weight ratio of rat lungs (n = 6, F = 23.200, *p* < 0.001). **(D)** Total protein concentration (F = 22.923, *p* < 0.001) and **(E)** Total cells count (F = 71.472, *p* < 0.001) in BALF (n = 6). **(F)** Representative images of IF staining of CD11b (green) and MPO (red) of different groups (scale: 50 μm, magnification ×400, n = 4). **(G)** Quantitative analysis of CD11b and MPO double-positive cells (F = 89.167, *p* < 0.001) in IF images. **(H, I)** TNF-α (F = 70.522, *p* < 0.001) and IL-1β (F = 91.517, *p* < 0.001) levels in BALF (n = 6). **(J–M)** TNF-α (F = 37.081, *p* < 0.001), IL-1β (F = 29.550, *p* < 0.001), vWf (F = 49.972, *p* < 0.001), and ET-1 (F = 45.677, *p* < 0.001) levels in serum (n = 6). Data are shown as mean ± SD; *p < 0.05, **p < 0.01, ***p < 0.001, ns no significance. W/D: Wet/Dry; BALF: bronchoalveolar lavage fluid; MPO: myeloperoxidase; IF: immunofluorescence; TNF: tumor necrosis factor; vWf: von Willebrand factor; IL: interleukin; ET-1: endothelin-1; SD: standard deviation.

The results of HE staining of lung tissues under light microscopy showed that the severity of lung injury in the groups receiving MV + SU6656 or MV + BP-1-102 was significantly lower than that in the group solely receiving MV (Figures 7A, B). The W/D ratio of lung tissue, total BALF protein concentration, and BALF cell count were also reduced in these groups (Figures 7C–E; Supplementary Figures S4A, B).

#### 3.3.2 Inhibitors of the Src/STAT3 pathway reduce inflammation response

A significant reduction was observed in MPO and CD11b double-positive cells in the lung tissues of the groups receiving MV + SU6656 and MV + BP-1-102 compared to those in the group solely receiving MV (Figures 7F, G). MPO and CD11b expressions decreased in the lung tissues of the groups receiving MV + SU6656 and MV + BP-1-102 compared to those in the group solely receiving MV (Supplementary Figures S4C–E). Furthermore, the groups treated with MV + SU6656 and MV + BP-1-102 showed significantly lower levels of TNF-α and IL-1β in BALF, and TNF-α, IL-1β, vWF, and ET-1 in serum compared to the MV group (Figures 7H–M).

#### 3.3.3 Inhibitors of Src/STAT3 reduce the pathway activation and pyroptosis without affecting PECAM-1

**FIGURE 8:**
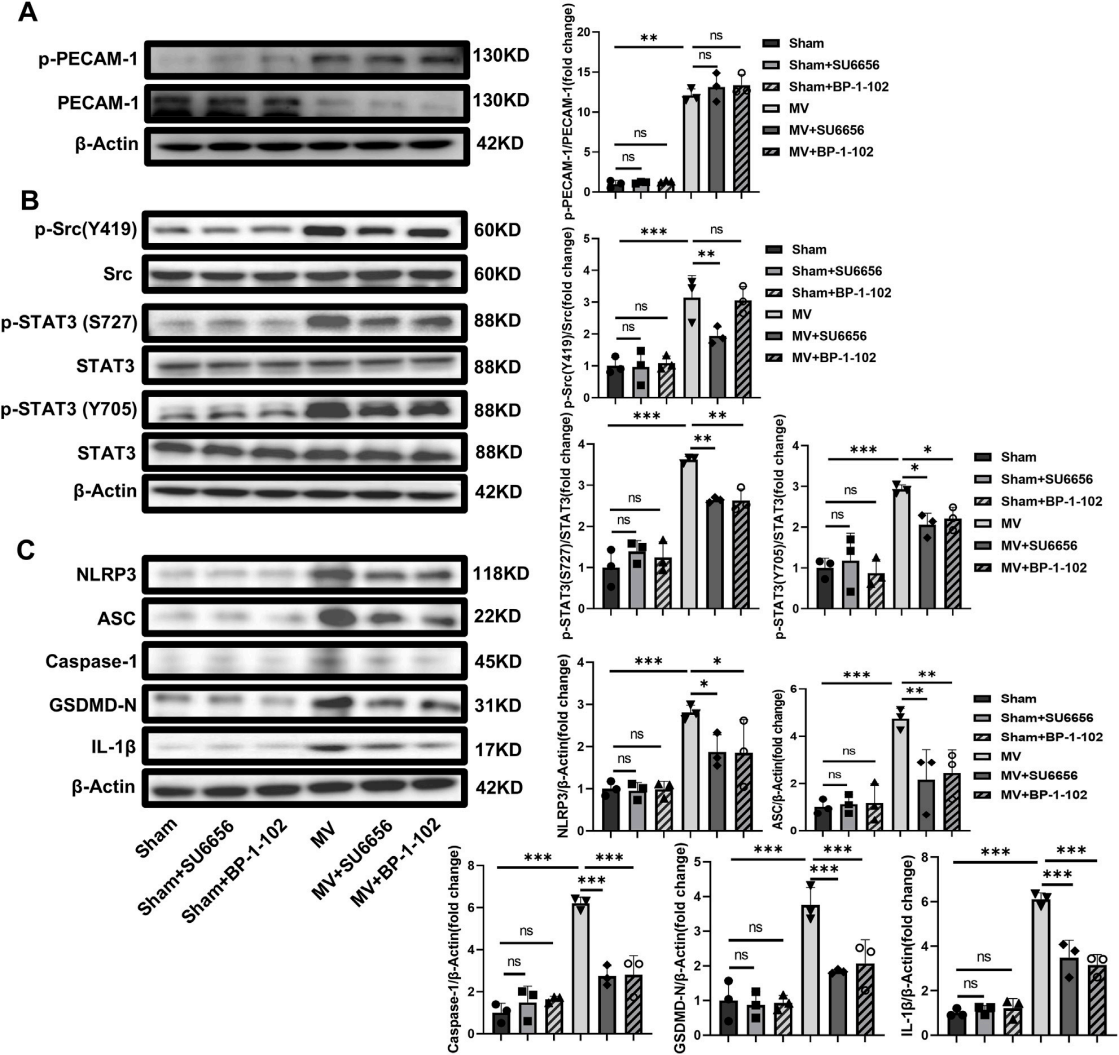
SU6656 and BP-1-102 Inhibit Src/STAT3 pathway activation and pyroptosis. **(A)** WB analysis of p-PECAM-1 and PECAM-1 in lung tissue (F = 127.920, *p* < 0.001). **(B)** WB analysis of p-Src, Src, p-STAT3, and STAT3 in lung tissue. p-Src (Y419)/Src (F = 16.524, *p* < 0.001), p-STAT3 (S727)/Src (F = 38.088, *p* < 0.001), p-STAT3 (Y705)/Src (F = 15.203, *p* < 0.001). **(C)** WB analysis of pyroptosis-related proteins in lung tissue, including NLRP3 (F = 10.802, *p* < 0.001), ASC (F = 9.855, *p* < 0.001), Caspase-1 (F = 32.298, *p* < 0.001), GSDMD-N (F = 17.704, *p* < 0.001), IL-1β (F = 61.028, *p* < 0.001). Data are shown as mean ± SD (n = 3). *p < 0.05, **p < 0.01, ***p < 0.001, ns no significance. PECAM-1: platelet endothelial cell adhesion molecule-1; STAT3: signal transducer and activator of transcription 3; WB: Western blot; SD: standard deviation.

The experiments indicated that SU6656 and BP-1-102 relieved VILI by inhibiting the activation of the Src/STAT3 pathway and decreasing the expression of pyroptosis-related proteins *in vivo* while the PECAM-1 phosphorylation remained unchanged (Figures 8A–C). These results demonstrated that Src/STAT3 signaling and pyroptosis contributed to VILI caused by UPLS.

## 4 Discussion

**FIGURE 9:**
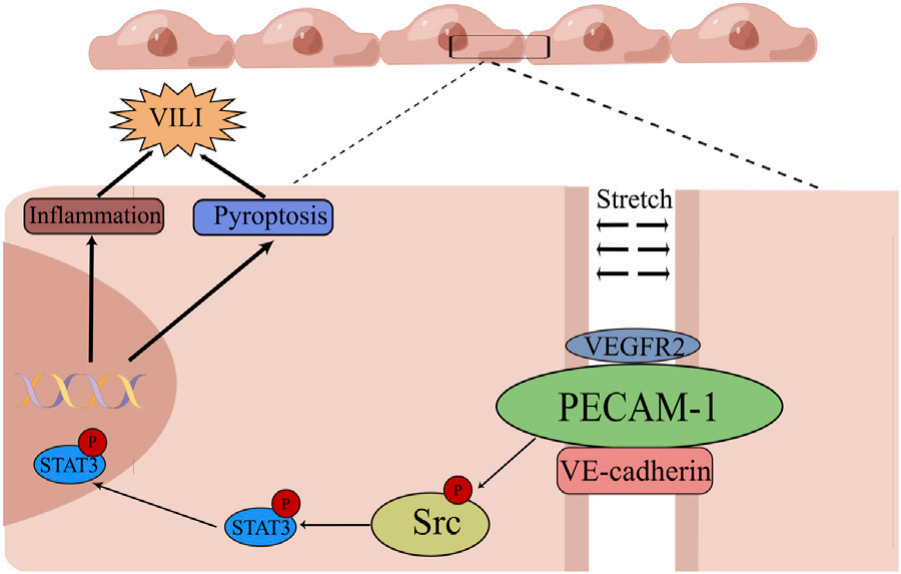
Graphical Abstract of the Mechanism of Unphysiological Lung Strain Promoting Ventilation-induced Lung Injury in this Study. Unphysiological lung strain can activate Src/STAT3 signaling via PECAM-1, leading to the subsequent translocation of p-STAT3 into the nucleus. p-STAT3 binds to the promoter region to transcriptionally modulate protein expression, leading to pyroptosis and a pulmonary inflammatory response, ultimately causing ventilation-induced lung injury (By Figdraw). PECAM-1: platelet endothelial cell adhesion molecule-1; STAT3: signal transducer and activator of transcription 3; VEGFR2: vascular endothelial growth factor receptor 2; VE-cadherin: vascular endothelial cadherin.

In recent decades, lung-protective ventilatory strategies using low Vt coupled with lung recruitment and high positive end-expiratory pressure (PEEP) have been adopted in clinical settings (Meade et al., 2008; Kacmarek et al., 2016). However, VILI still occurs in certain patients (Marini et al., 2020). Therefore, exploring the mechanism of VILI may contribute to finding novel treatment options for this complication. This study demonstrated that rats that exposed to MV with overlarge lung strain displayed a substantial lung injury, accompanied by phosphorylation of PECAM-1, Src/STAT3 pathways, and pyroptosis. Moreover, administering the PECAM-1 Ab and Src/STAT3 signal pathway inhibitors effectively alleviated pyroptosis and VILI in the rat model. The findings were validated through *in vitro* experiments with HUVECs exposed to mechanical cyclic stretch. Mechanistically, the PECAM-1/Src/STAT3 pathway and pyroptosis were involved in VILI caused by UPLS or overload mechanical CS, as shown in the graphical abstract (Figure 9).

Lung-protective ventilation strategies aim to prevent VILI primarily by controlling airway pressure or Vt. However, the plateau pressure and Vt during MV do not always accurately reflect the pressure on the lung tissue or the degree of change in the lung volume (Telias et al., 2022; Becher et al., 2021). Lung stress and strain are parameters in respiratory mechanics that more accurately mirror the changes in pressure and volume of lung tissue during MV (Mohammad et al., 2022). In this study, we adopted lung strain, the underlying trigger of VILI, as the reference to titrate Vt, which was the unique rationale. We found a significant increase in lung injury, pulmonary edema, and inflammatory responses when the lung strain value was increased to 1.5. This suggests that a lung strain value of 1.5 is the threshold at which VILI develops in Wistar rats. In studies with large animal models, VILI developed only when the global strain exceeded a critical interval between 1.5 and 2.0 (Protti et al., 2011; Liu et al., 2013). Small animals showed a weaker resistance to VILI than larger species, indicating a smaller safe threshold of lung strain than that in lager animals (Caironi et al., 2011), in line with the findings in this study. Additionally, our one-hit model demonstrates that excessive lung strain is an important etiological factor for VILI.

Lung inflammation is well-known to play an important role in the development of lung injury. Excessive lung strain and stress contribute to cellular inflammation, subsequently affecting VILI progression (Curley et al., 2016). Neutrophils accumulate in the interstitial and alveolar spaces and release injurious molecules, such as neutrophil elastase and metalloproteins, causing lung injury (Chen et al., 2018). MPO is expressed primarily in neutrophils and serves as functional and activation marker for neutrophils, whereas CD11b is primarily expressed on the cell membrane of neutrophils and monocytes and mediates leukocyte-endothelial cell interactions. The presence of CD11b and MPO double-positive cells suggests neutrophil infiltration (Ji et al., 2007). Inflammatory mediators including TNF-α and IL-1β are key modulators of inflammation that initiate and drive lung injury (Jin et al., 2020). In this study, the expression of endothelial dysfunction biomarkers such as vWF and ET-1, and CD11b and MPO double positive cells increased when lung strain reached 1.5, indicating that the UPLS provoked the inflammation.

However, the mechanism by which UPLS, an abnormality of respiratory mechanics, is transformed into biotrauma. PECAM-1, a critical component of the mechanosensing complex in adhesion junctions, may be affected first by the over-stretch during UPLS. The PECAM-1 cytoplasmic domain can be unfolded by externally applied forces, which enables rapid phosphorylation of endothelial cells when subjected to cyclic stretching (Snyder et al., 2017). Recirculation tensile strain (5%) caused a 42% increase in PECAM-1 phosphorylation (Meza et al., 2019). Phosphorylated PECAM-1 recruits Src homology 2 tyrosine/inositol phosphatases, thereby activating downstream signaling pathways, such as Src/STAT3 (Privratsky et al., 2010; Lertkiatmongkol et al., 2016; Arias et al., 2022). In this study, rats exposed to MV with UPLS experienced significant lung damage, with a notable increase in PECAM-1 phosphorylation in their lung tissues. Phosphorylation of PECAM-1 is the hallmark of PECAM-1 activation. Rats were treated with PECAM-1 Ab to further support our hypothesis that PECAM-1 played a significant role in this VILI model. We found that the phosphorylation of PECAM-1 was effectively blocked by PECAM-1 antibody, while its downstream Src/STAT3 signaling pathway was inhibited, and the degree of lung injury was significantly reduced, suggesting that the injection of our PECAM-1 antibody worked. These results further indicated that PECAM-1 activation played a crucial role in the progression of VILI caused by excessive pulmonary strain.

Src kinase is a vital component across multiple levels of the mechanosignalling network enabling cellular adaptation to mechanical forces (Heppner et al., 2018; Koudelková et al., 2021). Activated Src phosphorylates STAT3 thereby activating it, and activated STAT3 translocates to the nucleus and stimulates the transcription of a range of inflammatory factors (Kasembeli et al., 2018). The Src/STAT3 pathway was reported to be involved in inflammation and lung injury in an ischemia-reperfusion model (Oyaizu et al., 2012). We observed that Src/STAT3 signaling pathway activation occurred in the lung tissue when the lung strain value exceeded 1.5 *in vivo*. Conversely, we found the treatment with SU6656 and BP-1-102 inhibited the phosphorylation of Src and STAT3 respectively and reduced lung injury and inflammation; however, PECAM-1 phosphorylation was not affected. These findings suggest that the Src/STAT3 signaling pathway may aggravate VILI by activating inflammation. The PECAM-1 Ab decreased Src/STAT3 phosphorylation and relieved lung inflammation in this study; we considered that Src/STAT3 is an important downstream pathway of PECAM-1.

It is noteworthy that in the IF images, the Sham group also shows positive staining for MPO and CD11b, albeit not significantly, which was considered to be related to the operation procedures that the sham group underwent, including 4 h of tracheal intubation, which may induce inflammatory cell infiltration, even acute lung injury. This emphasizes the necessity of including a control group with tracheal intubation in studies investigating invasive MV as an intervention.

Besides inflammation, pyroptosis may be another damage mechanism mediated by PECAM-1/Src/STAT3. Pyroptosis is a type of programmed cell death that is mediated by the gasdermin family and plays a significant role in the pathogenesis of lung injury (Wang et al., 2021), which is accompanied by the release of pro-inflammatory cytokines such as IL-1β and IL-18 (Liu et al., 2016; Wei et al., 2022). Pyroptosis is mainly induced by the Caspase-1/GSDMD classical pathway (Yu et al., 2021; Feng et al., 2022a). The extent of pyroptosis can be evaluated by related proteins such as Caspase-1, NLRP3, and GSDMD (Feng et al., 2022a). In this study, the classical pyroptotic pathway, which involves NLRP3 inflammasomes and caspase-1 activation, was activated when lung strain reached 1.5. Administration of the PECAM-1 Ab and Src/STAT3 signaling pathway inhibitors inhibited pyroptosis, which suggested PECAM-1/Src/STAT3 and the resulting pyroptosis might play potential roles in the pathophysiology of VILI. This assumption has been validated in nonalcoholic steatohepatitis (Feng et al., 2022b).

The *in vitro* experiments with HUVECs presented the same findings, which confirmed the evidence was robust and the mechanism was reliable. Besides the PECAM-1, the other components (vascular endothelial-Cadherin and vascular endothelial growth factor receptor) of the mechanosensory complex can also be activated by shear stress and subsequently initiate the corresponding downstream pathways (Tzima et al., 2005; Fu et al., 2023),the findings in this study cannot rule out the potential impact of these pathways on the results.

This study had some limitations. First, we used a VILI model of MV in healthy rats rather than a “two-hit” model induced by LPS + MV to avoid the effects of lung injury resulting from other factors; however, the adopted one-hit model does not reflect the pathophysiological changes in most clinical patients with ARDS. Second, PECAM-1 triggered inflammatory responses and pyroptosis through the Src/STAT3 signaling pathway; however, the specific mechanism by which it induces pyroptosis remains to be determined. Thirdly, this study only focused on one mechanotransduction receptor, PECAM-1, although other receptors related to mechanotransduction such as piezo channels and transient receptor potential channels, may also be involved in VILI (Jia et al., 2022). Additionally, the cell damage by mechanical stretch was evaluated in this study by a widely used method, LDH cytotoxicity assay. However, the characteristic alignment of HUVECs perpendicular to the stretch axis was not reported in this part of *in vitro* experiments although it is an important indicator to evaluate the effect of stretch (Russo et al., 2018).

In conclusion, our findings indicate that PECAM-1 senses UPLS or overload CS and stimulates the Src/STAT3 signaling pathway, which in turn provokes pyroptosis and lung inflammatory response, ultimately leading to VILI. Furthermore, VILI would occur when lung strain reached 1.5 in Wistar rats, indicating that the safe threshold is smaller for smaller animals than for larger animals.

## Data Availability

The datasets presented in this study can be found in online repositories. The names of the repository/repositories and accession number(s) can be found in the article/Supplementary Material.

## Supplementary material

SUPPLEMENTARY FIGURE S1 Inflammatory cells in BALF and IHC staining of lung tissue. **(A, B)** Counts of Neutrophils (F = 71.925, P *<* 0.001) and Macrophages (F = 157.138, P *<* 0.001) in BALF. **(C)** Representative images of IHC staining of CD11b and MPO of Experiment 1 (scale: 100 μm, magnification ×200, n = 4). **(D, E)** Quantitative analysis plots of IHC images of Experiment 1 (MPO F = 19.863, P < 0.001, CD11b F = 46.957, P < 0.001). Data are shown as mean ± SD. **P <* 0.05, ***P <* 0.01, ****P <* 0.001, ns no significance. BALF: bronchoalveolar lavage fluid; IHC: Immunohistochemistry; MPO: myeloperoxidase; CD11b: Clusters of differentiation molecule 11b; SD: standard deviation.

SUPPLEMENTARY FIGURE S2 The reactivity of PECAM-1 Ab in the lung tissue of Rat and mouse as well as HUVEC **(A)** The reactivity of PECAM-1Ab to PECAM-1 in both C57BL/6 mouse and Wistar rat lung tissues by WB; **(B)** The reactivity of PECAM-1Ab to PECAM-1 in lung tissue of C57BL/6 mouse and Wistar rat (n = 2) as well as HUVECs by WB. WB: Western blot.

SUPPLEMENTARY FIGURE S3 Inflammatory cells in BALF and IHC staining of lung tissue after adopting PECAM-1 Ab **(A, B)** Counts of Neutrophils (F = 40.639, P < 0.001) and Macrophages (F = 52.759, P < 0.001) in BALF. **(C)** Representative images of IHC staining of CD11b and MPO (scale: 100 μm, magnification ×200, n = 4). **(D, E)** Quantitative analysis plots of IHC images (MPO F = 28.654, P < 0.001, CD11b F = 27.336, P < 0.001). Data are shown as mean ± SD. **P <* 0.05, ***P <* 0.01, ****P <* 0.001, ns no significance. BALF: bronchoalveolar lavage fluid; IHC: Immunohistochemistry; MPO: myeloperoxidase; CD11b: Clusters of differentiation molecule 11b; SD: standard deviation.

SUPPLEMENTARY FIGURE S4 Inflammatory cells in BALF and IHC staining of lung tissue after inhibiting Src/STAT3. **(A, B)** Counts of Neutrophils (F = 165.591, P < 0.001) and Macrophages (F = 103.181, P < 0.001) in BALF. **(C)** Representative images of IHC staining of CD11b and MPO (scale: 100 μm, magnification ×200, n = 4). **(D, E)** Quantitative analysis plots of IHC images (MPO F = 39.844, P < 0.001, CD11b F = 40.555, P < 0.001). Data are shown as mean ± SD. **P <* 0.05, ***P <* 0.01, ****P <* 0.001, ns no significance. BALF: bronchoalveolar lavage fluid; IHC: Immunohistochemistry; MPO: myeloperoxidase; CD11b: Clusters of differentiation molecule 11b; SD: standard deviation.
