# Supplementary figures and images for: Unphysiological lung strain promotes ventilation-induced lung injury via activation of the PECAM-1/Src/STAT3 signaling pathway
Source: Front Pharmacol. 2025 Jan 8;15:1469783. doi: 10.3389/fphar.2024.1469783 (PMC11751019; doi:10.3389/fphar.2024.1469783)

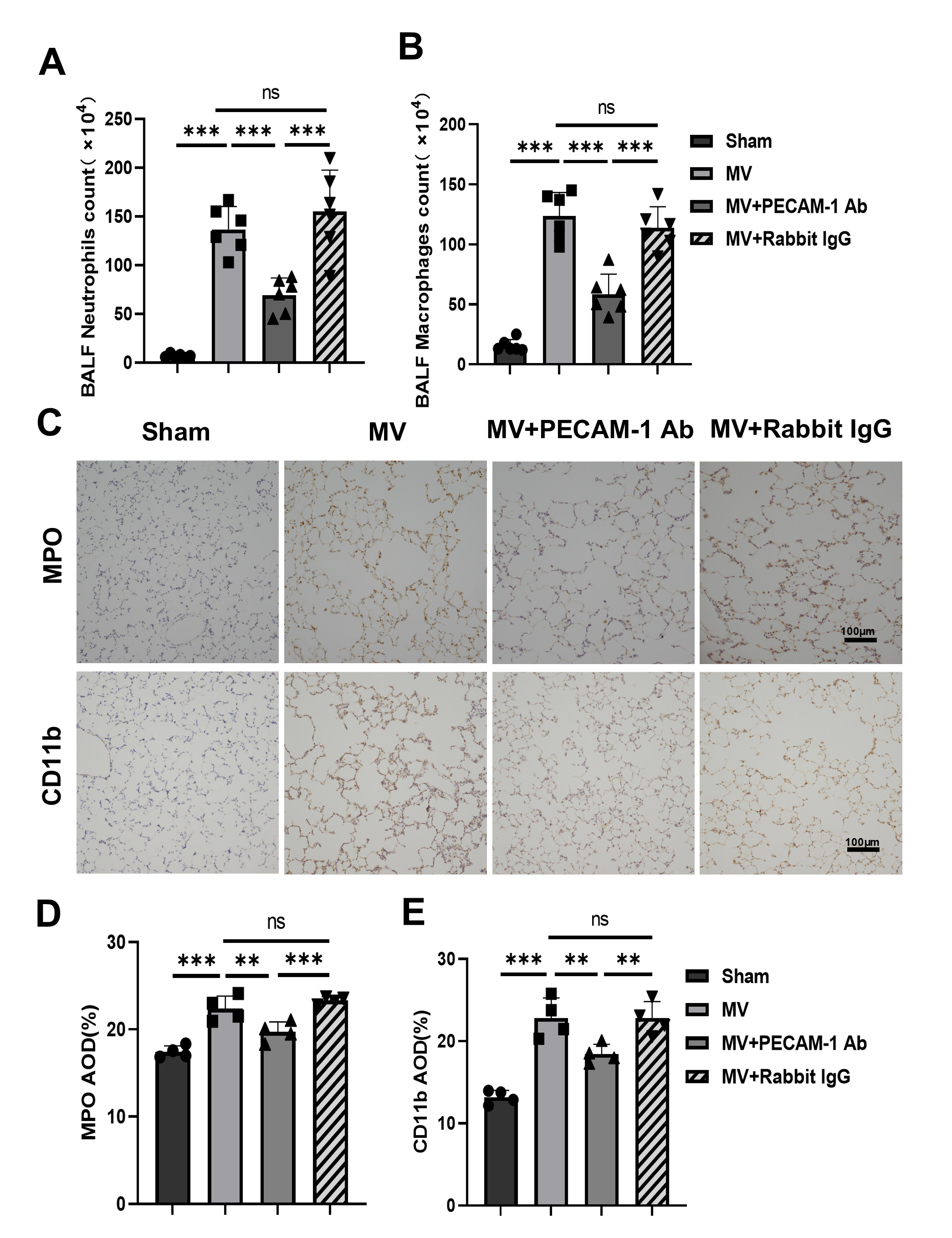

Supplement: Supplementary file 2 [file Image3.tif]

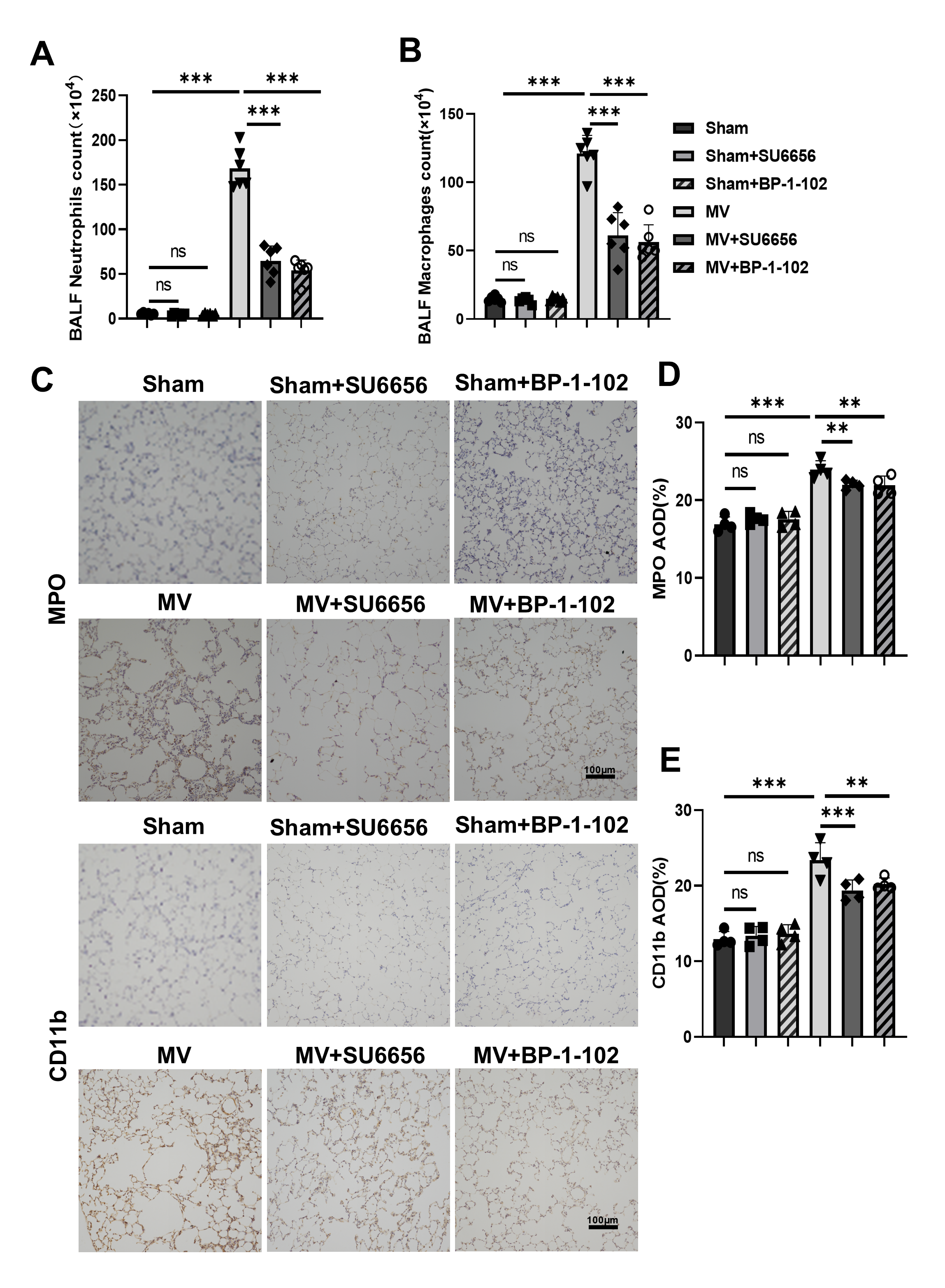

Supplement: Supplementary file 3 [file Image4.tif]

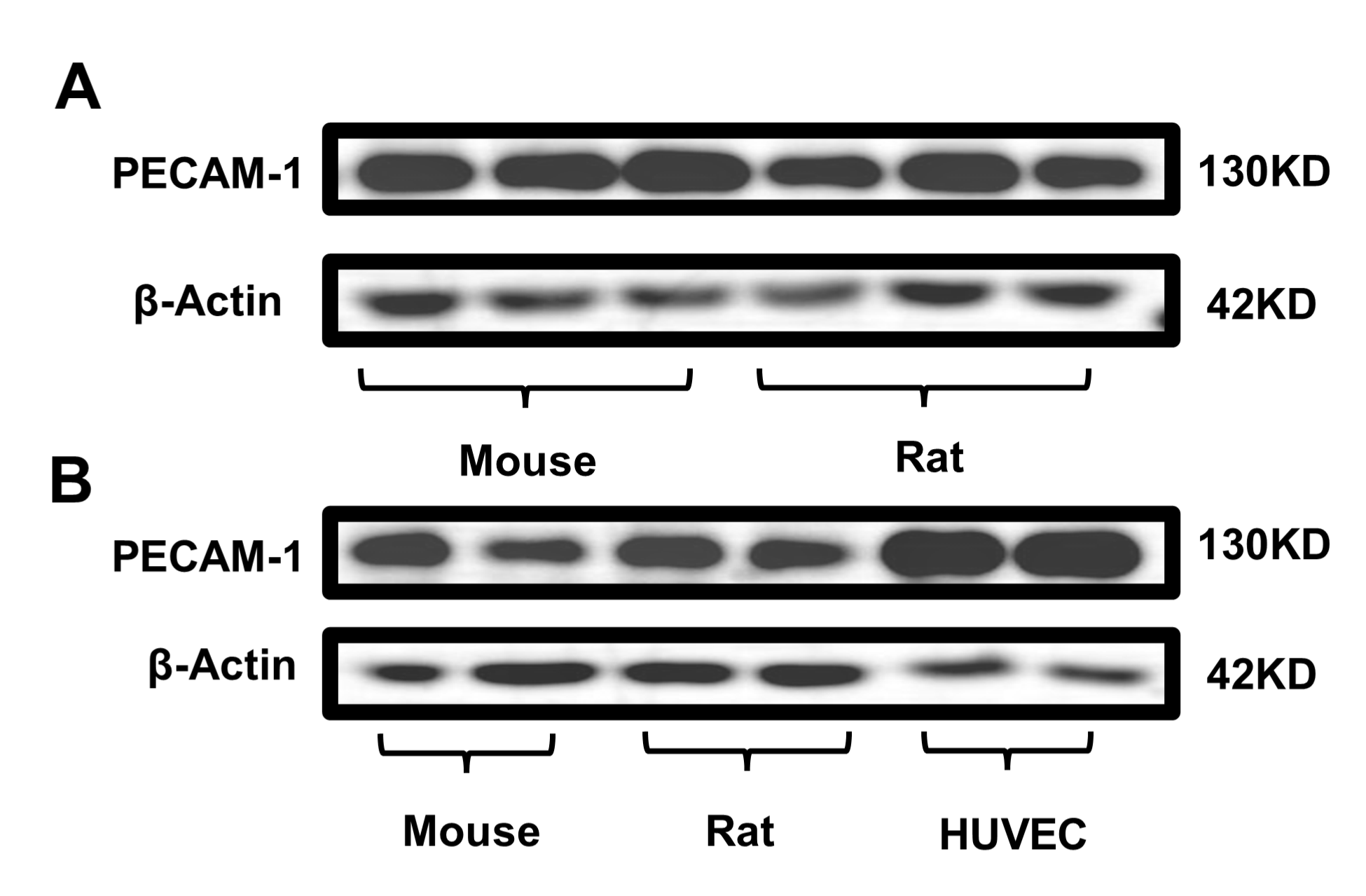

Supplement: Supplementary file 4 [file Image2.tif]

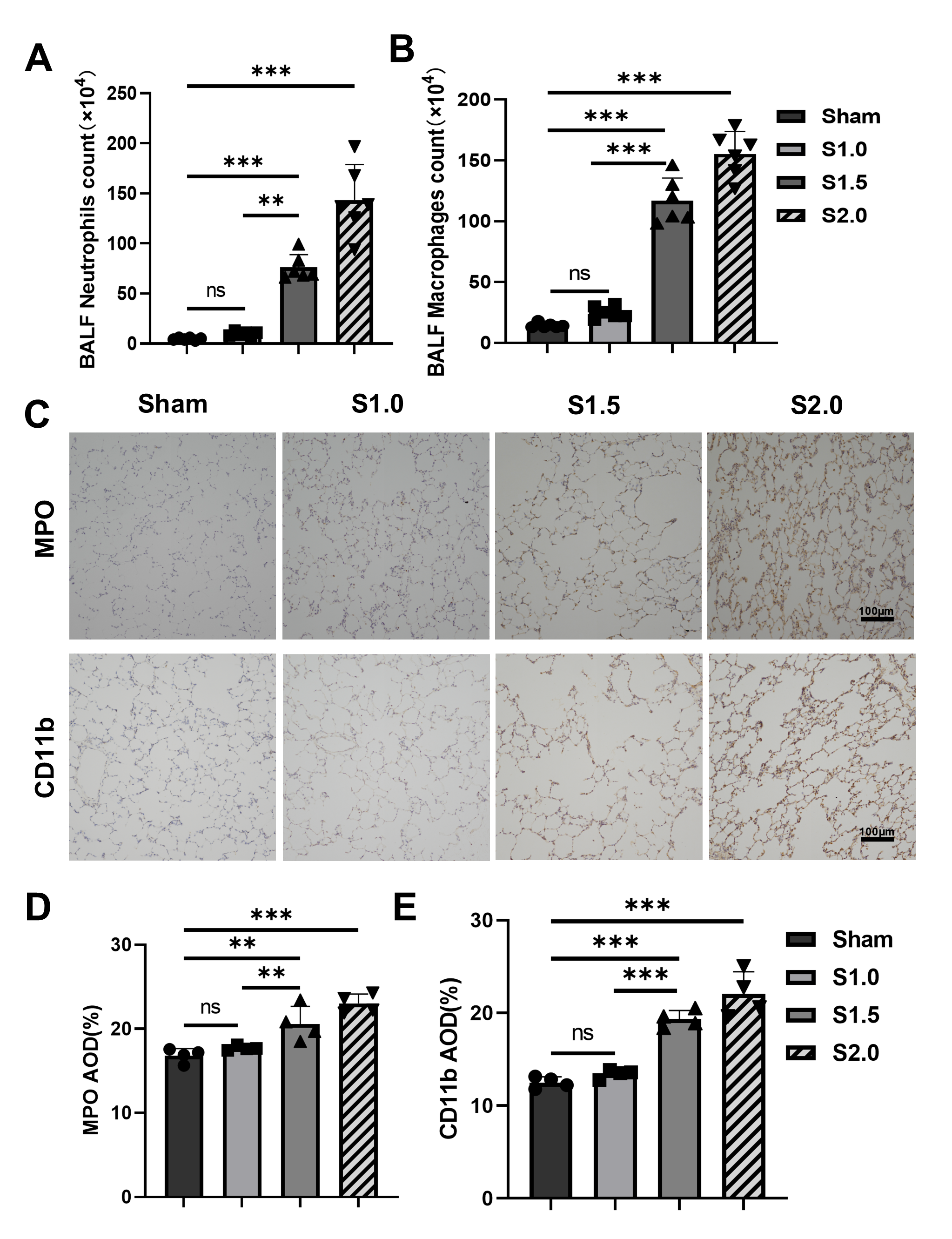

Supplement: Supplementary file 5 [file Image1.tif]
