# Supplementary material for: Unphysiological lung strain promotes ventilation-induced lung injury via activation of the PECAM-1/Src/STAT3 signaling pathway
Source: Front Pharmacol. 2025 Jan 8;15:1469783. doi: 10.3389/fphar.2024.1469783 (PMC11751019; doi:10.3389/fphar.2024.1469783)
Supplement: Supplementary file 6 [file Table1.doc]

**Supplementary Materials**

**Table S1: Key information of reagents used in the experiments**

| **Name of the reagent** | **Company** | **City** | **Country** |
| --- | --- | --- | --- |
| Sodium pentobarbital | Sigma-Aldrich | St. Louis, Missouri | USA |
| PECAM-1 Ab | BioLegend | San Diego, CA | USA |
| Src inhibitor SU6656 | Selleck | Shanghai | China |
| STAT3 inhibitor BP-1-102 | Selleck | Shanghai | China |
| Rabbit IgG | Proteintech | Wuhan | China |
| BCA assay kit | Solarbio Science and Technology | Beijing | China |
| Wright-Giemsa stain | Solarbio Science and Technology | Beijing | China |
| CCK-8 | NCM Biotech | Suzhou | China |
| LDH Cytotoxicity Assay Kit | Beyotime Biotechnology | Nanjing | China |
| TNF-α ELISA kits | Thermo Fisher Scientific | Waltham, Massachusetts | USA |
| IL-1β ELISA kits | MultiSciences Biotech | Hangzhou | China |
| von Willebrand factor(vWF) | Cusabio | Wuhan | China |
| endothelin-1(ET-1) ELISA kit | Cusabio | Wuhan | China |
| HRP conjugated rabbit anti-mouse IgG polyclonal antibody | Proteintech | Wuhan | China |
| HRP conjugated rabbit anti-mouse IgG polyclonal antibody | Huabio | Hangzhou | China |
| Cy3-labeled Goat Anti-Rat IgG (H+L) | Beyotime Biotechnology | Nanjing | China |
| Alexa Fluor 488-labeled Goat Anti-Rabbit IgG (H+L) | Beyotime Biotechnology | Nanjing | China |
| 4',6-diamidino-2-phenylindole (DAPI) | Beyotime Biotechnology | Nanjing | China |
| RIPA buffer | Epizyme Biomedical Technology | Shanghai | China |
| Anti-CD11b | Servicebio | Wuhan | China |
| Anti-MPO | Servicebio | Wuhan | China |
| Anti-p-Src (phospho Y527) | CST | Danvers, MA | USA |
| Anti-p-STAT3 (phosphor Y705) | CST | Danvers, MA | USA |
| Anti-IL-1β | CST | Danvers, MA | USA |
| Anti-GSDMD-N | CST | Danvers, MA | USA |
| Anti-STAT3 | Proteintech | Wuhan | China |
| Anti-PECAM-1 | Proteintech | Wuhan | China |
| Anti-NLRP3 | Proteintech | Wuhan | China |
| Anti-ASC | Proteintech | Wuhan | China |
| Anti-Caspase-1 | Proteintech | Wuhan | China |
| Anti-β-actin | Proteintech | Wuhan | China |
| Anti-PECAM-1([phospho-Y713](https://www.biorbyt.com/pecam1-phospho-y713-antibody-orb571507.html)) | Biorbyt Ltd | Cambridge | UK |
| Anti-Src | Abcam | Cambridge | UK |
| Anti-p-Src (phospho Y419) | Abcam | Cambridge | UK |
| Anti-p-STAT3 (phospho S727) | Abcam | Cambridge | UK |
| polyvinylidene fluoride membranes | Millipore | MA | USA |
